# Physio-Biochemical Composition and Untargeted Metabolomics of Cumin (*Cuminum cyminum* L.) Make It Promising Functional Food and Help in Mitigating Salinity Stress

**DOI:** 10.1371/journal.pone.0144469

**Published:** 2015-12-07

**Authors:** Sonika Pandey, Manish Kumar Patel, Avinash Mishra, Bhavanath Jha

**Affiliations:** 1 Division of Marine Biotechnology and Ecology, CSIR-Central Salt and Marine Chemicals Research Institute, G. B. Marg, Bhavnagar (Gujarat), India; 2 Academy of Scientific and Innovative Research, CSIR, New Delhi, India; Hainan University, CHINA

## Abstract

Cumin is an annual, aromatic, herbaceous, medicinal, spice plant, most widely used as a food additive and flavoring agent in different cuisines. The study is intended to comprehensively analyse physiological parameters, biochemical composition and metabolites under salinity stress. Seed germination index, rate of seed emergence, rate of seed germination, mean germination time, plant biomass, total chlorophyll and carotenoid contents decreased concomitantly with salinity. In contrast, total antioxidant activity, H_2_O_2,_ proline and MDA contents increased concurrently with stress treatments. Total phenolic and flavonoid contents were decreased initially about 1.4-fold at 50 mM, and thereafter increased about 1.2-fold at 100 mM NaCl stress. Relative water content remained unchanged up to 50 mM NaCl stress, and thereafter decreased significantly. About 2.8-fold electrolyte leakage was found in 50 mM, which increases further 4-fold at 100 mM NaCl stress. Saturated fatty acids (FAs) increased gradually with salinity, whereas unsaturation index and degree of unsaturation change arbitrarily along with the percent quantity of unsaturated FAs. Total lipid and fatty acid composition were significantly influenced by salinity stress. A total of 45 differentially expressed metabolites were identified, including luteolin, salvianolic acid, kaempferol and quercetin, which are phenolic, flavonoid or alkaloids in nature and contain antioxidant activities. Additionally, metabolites with bioactivity such as anticancerous (docetaxel) and antimicrobial (megalomicin) properties were also identified. The study evidenced that plant shoots are a rich source of metabolites, essential amino acids, phenolic compounds and fatty acids, which unveil the medicinal potential of this plant, and also provide useful insight about metabolic responses under salinity stress.

## Introduction

Cumin (*Cuminum cyminum* L.) is a small, aromatic, annual, diploid cross pollinated herb of the family Apiaceae. It is cultivated in arid and semi-arid areas, including India, Middle East, China and Mediterranean region. The plant is an active reservoir of numerous bioactive compounds with various therapeutic applications [1–2]. It is globally popular and essential for flavoring in many cuisines, particularly South Asian, Northern African and Latin American cuisines. Cumin seeds are used as a spice for their distinctive flavor and aroma. It enhances the appetite, taste perception, digestion, vision, strength and lactation. It is also used in the treatment of fever, loss of appetite, diarrhea, vomiting, abdominal distension, edema and puerperal disorders [2–4]. Thus cumin seeds are of considerable importance because of its nutritional values and other health benefits. Dried cumin seeds contain volatile oil (5%), fat (22%), protein (10%), fibre (11%) and free amino acids [5]. The characteristic aroma of volatile oil, obtained from dried cumin seeds are attributed to the presence of 3p-menthen-7al, β–pinene, p-cymene, γ-terpinene, p-mentha-1, 3-dien-7-al, p-mentha-1 and cuminaldehyde in combination with other related aldehydes [6]. Cuminaldehyde, cymene and terpenoids are the major volatile components of cumin. Cuminaldehyde has also antimicrobial and antifungal properties, which could be shown with *Escherichia coli* and *Penicillium chrysogenum* [2]. The anti-carcinogenic activity has also been studied and cumin seeds are found potent inhibitor of both squamous cell carcomas and hepatomas [7]. In herbal medicine, cumin oil is known to possess several pharmacological activities, such as antimicrobial, anti-diabetic, antiepileptic, anti-infertility, anticancerous and immunomodulative effects due to presence of active chemical constituents. Aqueous or solvent extract of cumin is known to inhibit growth of many pathogenic micro-organisms [1, 8–9].

Soil salinity is ever-present abiotic factor responsible for yield and productivity loss of domesticated crops worldwide. Nearly more than one-third of arable land of the world is affected by salinity [10]. Cumin is salt sensitive plant and its growth is drastically reduced with increasing salinity stress [11]. Physiologically, cumin plant has ability to tolerate 5 dS m^-1^ of salt in irrigated water but its seed productivity is significantly reduced by 55% [11–12].

Cumin plant frequently encounters with various environmental stresses, which endorse positive effect on plant physiology and biosynthesis of secondary metabolites as such responses may be necessary to develop tolerance against constraining condition for survivability [13]. Antioxidant activities, synthesis of metabolites and physiological status of plants are influenced by varying stress conditions. Metabolic pathways are highly dynamic and metabolites are low-molecular-weight osmolytes that play a key role in osmotic adjustment. Comprehensive analyses of biochemical composition and metabolites (primary and secondary) under environmental stress exhibit new responses and thus provide the physiological status of a plant. Nowadays, different analytical methods have been developed to elucidate comprehensive information about plant physiology, biochemical composition and metabolites by comparative homology-based analyses [14]. Till date, no information is available on physio-biochemical composition and metabolomics of this important spice plant. Therefore, physiological, biochemical and metabolite compositions of cumin shoot were studied and compared with salinity stress conditions. The study provides useful comprehension of the cumin plant to be used as functional food and also metabolic responses to alleviate salinity stress.

## Materials and Methods

### Plant samples, stress treatments and seed germination assay

Mature seeds of cumin (local variety GC-3) were surface sterilized using 2% (v/v) sodium hypochlorite for 10 minutes, later on rinsed five times with autoclaved distilled water and kept overnight in water [15–16]. Experiments were divided into two sets.

In the first experiment, seed germination, emergence rate and morphological parameters of cumin seeds were studied under different NaCl concentrations. Seeds were placed on filter paper for germination in petri dishes, supplemented with different concentrations of NaCl (0, 30, 50, 80 and 100 mM) and kept in the dark incubation at 21°C up to emergence of radicals. Germination test was conducted in four replicates of 25 seeds. Mean germination time of seeds was recorded up to 15 days and seeds were counted after every 24 hrs. Seeds with emergence of radicle (at least 2 mm) were considered as germinated [17]. Rate of emergence or rate of germination [18], mean germination time (MGT) [19] and germination index (GI) [20] were calculated with the following formula:

$$Rate\;of\;emergence\;or\;germination=\frac{n1}{t1}+\frac{n2}{t2}+\cdots \;\frac{n15}{t15}$$

where n1, n2, …n15 are the number of emerged/ germinated seeds at times t1, t2, …t15 (in days)

$$Mean\;germination\;time\;(MGT)=\frac{\Sigma (fx)}{\Sigma f}$$

where *f* is the number of newly germinated seeds on each day and *x* is days of counting.

$$GI=\frac{Germination\;\%\;in\;each\;treatment}{Germination\;\%\;in\;the\;control}\times 100$$

After 20 days of complete emergence of radicle and plumule, length and fresh weight (of root and shoot) were recorded. Later on seedlings were harvested and dried in oven for 48 h at 70°C for measurement of dry weight.

In second set of experiment, sterilized seeds of cumin were germinated on basal salt (MS) [21] in culture bottles. The MS medium was supplemented 3% (w/v) sucrose and 0.8% (w/v) agar and pH of the medium was adjusted to 5.8 prior to autoclaving. Germinated seedlings (15 days old) of uniform size were transferred to hydroponic culture in ½ of MS salt nutrient medium and grown under laboratory conditions with light/dark cycle of 16/8 h at 24 ± 2°C. After 10 days, plants were subjected to NaCl salt treatment of 50 mM and 100 mM, whereas nutrient solution without NaCl was taken as control. The nutrient solution was replaced at 5 days interval. After seven days of salt treatment, plant samples were harvested for physiological and biochemical analyses.

### Photosynthetic pigment extraction and measurement

Fresh leaves (100 mg) were ground in chilled N, N-dimethylformamide (DMF) at 4°C with mortar and pestle in dark. The homogenate was centrifuged at 10,750 g for 15 min at 4°C to remove cell debris. Supernatants were collected and absorbance was recorded at 665, 647 and 461 nm using a UV–Visible spectrophotometer (SpectramaxPlus 384, Molecular Devices, USA). Different photosynthetic pigments, such as chlorophyll a (Chl a), chlorophyll b (Chl b), total chlorophylls (Chl a+b) and carotenoid were quantified [22–23].

### Analysis of primary metabolites

#### Amino acid composition

Total protein was extracted from shoots of both control and salt treated samples (1 g) using trichloroacetic acid (TCA) precipitation method [24], and concentration was determined by Bradford assay [25]. Hydrolysis of total extracted protein was carried out in glass tube with HCl (6 N, 500 μl) at 110°C for 24 h. On the completion of hydrolysis, protein samples and amino acid standard (AAS18, Sigma, St. Louis, Missouri, USA) were treated with mixture of ethanol–water–TEA (2:2:1, v/v/v; 500 μl) for neutralization. Samples were mixed, vacuum dried and subjected to the derivatization [26] by adding a mixture of ethanol–water–TEA–PITC (7:1:1:1, v/v/v/v; 50 μl). The reaction mix was incubated at room temperature for 20 min to produce phenylthiocarbamyl amino acids. Samples were vacuum dried and dissolved in phosphate buffer (5 mM, pH 7.4; 400 μl), containing acetonitrile (5%, v/v). Samples, after filtration with a 0.2 μm membrane were used for determination of amino acid composition using HPLC (Waters Alliance model, 2996-seperation module with autosampler), equipped with Luna-C18 reversed-phase (5.0 μm, 4.6 × 150 mm, Phenomenex, Torrance, California, USA) column [26–27]. The amino-acid composition was determined by calculating the relative proportion of peak area [14].

#### Fatty acid extraction and analysis

Total lipid was extracted from shoot samples (250 mg), grown under different salinity stress using chloroform–methanol–phosphate buffer (1:2:0.9, v/v/v, pH 7.5; 10 ml) following modified Bligh and Dyer method [28]. Extracted fatty acids were converted to their corresponding fatty acid methyl esters (FAMEs) by transmethylation [14]. The derivatized FAMEs were extracted in hexane and analyzed on GCMS–QP2010 (Shimadzu, Kyoto, Japan) equipped with an auto-sampler (AOC-5000) using a RTX 5MS capillary column [14]. FAME peaks were identified by comparing with standards (FAME Mix C4-C24, Supelco, Bellefonte, Pennsylvania, USA) run in GC–MS along with samples and represented as percent quantity. Saturated fatty acids (SFA), unsaturated fatty acids (monounsaturated fatty acids, MUFA and polyunsaturated fatty acids, PUFA) were calculated by summation of percent quantity of corresponding fatty acids, whereas unsaturation index [29], degree of unsaturation [30], Atherogenic and thrombogenic indices [31] were calculated using the following formula:

Degreeofunsaturation(DU)=(MUFAw%)+2×(PUFAw%)

Unsaturationindex(UI)=Σ(UFAw%×numberofdoublebonds)

$$Atherogenic\;index\;(AI)=\frac{C12+C14+C16}{\Sigma n3PUFA+\Sigma n6PUFA+\Sigma MUFA}$$

$$Thrombogenic\;index\;(TI)=\frac{C14+C16+C18}{\left(0.5\times n6PUFA)+(3\times n3PUFA)+(n3/n6PUFA\right)}$$

#### Total soluble sugar

Plant shoot sample was homogenized and total sugar was extracted in aqueous alcohol (80%, v/v). Total soluble sugar was quantified using Anthrone reagent (0.2%, w/v anthrone in concentrated H_2_SO_4_) and glucose (0–1000 mg l^-1^) was used as standard curve. The alcoholic extract (0.25 ml) was mixed with 3 ml of Anthrone reagent and incubated at 100°C for 10 min. The solution cooled at room temperature, absorbance was recorded at 630 nm and total soluble sugar estimated from the standard curve.

### Secondary metabolites profiling

#### Total phenolic content

The concentration of total phenolic content of plant extract was determined by using Folin–Ciocalteu reagents [32]. The plant extract was mixed with 2.5 ml of 0.2 M Folin–Ciocalteu reagents (Sigma, St. Louis, Missouri, USA) and incubated for 5 min. The reaction was neutralized with saturated sodium carbonate solution (2 ml, 75 g l^-1^). Reaction mixtures were incubated for 90 min at room temperature, absorbance was read at 760 nm and total phenolic content was evaluated from a gallic acid standard curve (mg ml^−1^ gallic acid per 100 mg of extract).

#### Total flavonoid content

Total flavonoid content of plant extract was determined as described previously [32]. Plant extract was mixed with 0.3 ml NaNO_2_ (5%, v/v). Reaction mixture was allowed to stand for 5 min at room temperature followed by addition of 0.3 ml AlCl_3_ (10%, v/v) and later on 2 ml NaOH (1 M). Finally, reaction mixture was diluted and absorbance was recorded at 510 nm. Standard curve of quercetin was used for calculation of total flavonoid and expressed as mg ml^−1^ quercetin per 100 mg of extract.

### Extraction and analysis of metabolites

Total plant metabolites were extracted and analyzed by LC MS [14, 33]. Plant shoot sample (200 mg) was homogenized in liquid N_2_ and total metabolites were extracted in ice cold methanol (70%, v/v) followed by sonication in ultrasonic water bath (MRC, Holon, Israel) for 1 h at frequency 40 kHz (25°C). Sample was centrifuged at 18,000 g for 10 min at 25°C, supernatant was filtered through 0.2 μm membrane and used for metabolites identification. Analysis of compounds was done by LC coupled with TOF-MS (Micromass, Waters, Milford, Massachusetts, USA) and identified by comparing LC-TOF-MS peaks using on-line METLIN database [34].

### Physiological analysis

#### Hydrogen peroxide activity

Leaf tissues were homogenized in 5 ml chilled acetone. Supernatant (200 μl) obtained after centrifugation was mixed with reaction buffer that contains 0.25 mM FeSO_4_, 0.25 mM (NH_4_)_2_SO_4_, 25 mM H_2_SO_4_, 1.25 mM xylenol orange and 1 mM sorbitol. Reaction mixture after incubation at 25°C for 30 min was used for the quantification of H_2_O_2_ at 560 nm absorbance [35–36].

#### Proline extraction and quantification

Free proline content of harvested leaf sample was extracted using 3% (w/v) sulphosalicylic acid and determined by acid ninhydrin reagent [37]. Leaf tissue was homogenized in 2 ml of aqueous sulphosalicylic acid and centrifuged at 10,750 g for 15 min at 4°C. The supernatant (1 ml) was mixed with 1 ml of each acid ninhydrin and glacial acetic acid followed by incubation at 98°C for 1 h. Reaction mixture was cooled on ice cold water and 2 ml of toluene was added followed by vortexing. Upper phase was separated and absorbance was recorded at 520 nm against blank.

#### Lipid peroxidation quantification

Lipid peroxidation was calculated by determining the concentration of malondialdehyde (MDA) using method as described by Hodges *et al*. [38]. Leaf sample was ground in 1 ml of 0.1% (w/v) TCA reagent. The homogenate was centrifuged for 10 min at 18,000 g to get the clear supernatant. Two set of samples were prepared for the experiment. In 1^st^ set of experiment extracted plant sample (0.5 ml) was mixed with 2 ml TBA (0.65%, w/v; prepared in TCA 20%, w/v), whereas in 2^nd^ set sample (0.5 ml) was mixed with 20%, w/v TCA. Reaction mixtures were incubated at 95°C for 30 min, thereafter centrifuged at 10,750 g for 5 min. Collected supernatant was used for measurement of absorbance at 440 nm, 532 nm and 600 nm. MDA content was quantified by using following equations:

$$A=({Abs}_{532+TBA}-{Abs}_{600+TBA})-({Abs}_{532-TBA}-{Abs}_{600-TBA})$$

$$B=({Abs}_{440+TBA}-{Abs}_{600-TBA})\times 0.0571$$

$$MDA(nmol\;g^{-1})=\frac{\left(\frac{A-B}{157000}\right)\times 10^{6}}{Weight\;of\;tissues\;in\;gram}$$

#### Total antioxidant activity

Plant shoot samples were homogenized in liquid N_2,_ mixed with 70%, v/v aqueous methanol and kept overnight on stirring. Supernatant was collected after centrifugation at 5,250 g for 15 min. The antioxidant activity was measured as ABTS (2, 2′-azinobis-(3-ethylbenzothiazoline-6-sulfonic acid) cation decolorization assay [14, 39]. The ABTS solution (7 mM) was made to react with potassium persulfate (2.45 mM) in dark at room temperature for 12–16 h to generate ABTS free radical cations before use. The absorbance of the ABTS^.+^ solution was adjusted to 0.70 ± 0.02 by diluting with water at room temperature. Plant extract with different concentrations (50–200 μl) was mixed with 1 ml of diluted radical cation solution and absorbance was recorded at 734 nm after incubation. The percentage inhibition of absorbance was calculated and activity was compared with trolox as standard.

#### DPPH Inhibition

DPPH (2, 2-diphenyl-1-picrylhydrazyl) scavenging assay was performed by measuring change in the color of DPPH solution (at 517 nm) due to radical scavenging activity of antioxidant compound from deep violet to pale yellow non-radical form of DPPH-H [40]. Stock solution of DPPH (0.024%, w/v in methanol) was diluted by adding methanol to obtain an absorbance of about 0.98 ± 0.02 at 517 nm. Reaction mixture, containing different concentrations of plant extract and diluted DPPH solution (3 ml) were incubated in dark at room temperature for overnight. Later on the radical scavenging activity of plant tissue extract was estimated as DPPH radical scavenged using the following equation:

$$Scavenging\;activity(\%)=\left(\frac{{OD}_{517}\;of\;control-{OD}_{517}\;of\;extract}{{OD}_{517}\;of\;control}\right)\times 100$$

#### Relative water content

Fresh leaves about 2 cm long were weighed (FW) and kept immersed in deionized water at room temperature for 8 h. Leaves were blotted dry and turgid weight (TW) was recorded. After that, samples were wrapped in pre weighed foil and kept at 80°C for 48 h in oven for drying to get the dry weight (DW). Leaf relative water content was measured [41] and expressed as percentage by the following formula:

$$RWC(\%)=\left(\frac{FW-DW}{TW-DW}\right)\times 100$$

Where FW, DW and TW are fresh weight, dry weight and turgid weight, respectively.

#### Electrolyte leakage

Fresh leaves excised from control and NaCl treated plants were washed in deionized water and cut into 2 cm segments. Samples were immersed in 10 ml deionized water and kept in gentle shaking for 4 h at room temperature. Electrolyte leakage was calculated [42] by measuring initial electrical conductivity (*L*_*t*_) and final electrical conductivity (*L*_*0*_) of bathing solutions (obtained by boiling at 99°C for 20 min) using conductivity meter (SevenEasy, Mettler Toledo AG 8603, Switzerland).

$$Electrolyte\;leakage(\%)=\left(\frac{L_{t}}{L_{0}}\right)\times 100$$

### Preparation of extract for enzyme activity and assay

Plant shoot samples (control and salt treated) were homogenized in liquid N_2_ and mixed with extraction buffer, containing 50 mM phosphate buffer solution (pH 7.0) and 0.5 mM EDTA. Homogenate was centrifuged at 10,750 g for 15 min at 4°C and supernatant was collected to measure activity of peroxidase (POD; EC 1.11.1.7), ascorbate peroxidase (APX; EC 1.11.1.11) and glutathione reductase (GR; EC 1.6.4.2).

Standard reaction mixture for POD activity assay contained phosphate buffer (1 ml, 0.05 M, pH 7.0), H_2_O_2_ (1 ml, 0.3%, v/v) guaiacol (0.95 ml, 0.2%, v/v) and 50 μl of enzyme extract in a final volume of 3 ml [43–44]. Absorbance was recorded immediately after addition of enzyme extract and after 3 min of incubation at 34°C at 470 nm (ɛ = 25.5 mM^-1^ cm^-1^).

For the measurement of APX activity, reaction mixture contained 50 mM phosphate buffer (pH 7.0), 0.1 mM EDTA, 0.5 mM ascorbate, 1.0 mM H_2_O_2_ and 50 μl enzyme extract [45]. The reaction rate for ascorbate oxidation was recorded at 290 and specific activity of enzyme was determined by molar extinction coefficient of ascorbate (2.8 mM^-1^ cm^-1^).

The GR activity assay solution was comprised of 100 mM phosphate buffer (pH 7.5), 0.5 mM EDTA, 0.75 mM DTNB, 0.1 mM NADPH and enzyme extract [46]. Reaction was started by the addition of 1.0 mM oxidized glutathione (GSSG). As a consequence, TNB (2-nitro-5-thiobenzoate) was produced by reduction of DTNB (5, 5-dithiobis (2 nitrobenzoic acid)) by glutathione (GSH) and increase in absorbance was recorded at 412 nm (ε = 06.22 mM^-1^ cm^-1^) to calculate specific activity of enzyme.

### Determination of ion content (ICP)

Tissue samples (root and shoot) were harvested and kept in a hot air oven at 70°C for 48 h for drying. Dried samples were digested with 5 ml mixture of nitric acid and perchloric acid (9:4, v/v) in 100 ml volumetric flask until the production of red NO_2_ fumes ceased. After that, solution containing digested plant material was evaporated until volume remained up to 1 ml. When the liquid became colorless, flask was cooled and deionized water was added to make final volume 25 ml. Subsequently digested solution was filtered (through Whatman No. 1 filter paper) and ion contents were measured by inductively coupled plasma atomic absorption spectrometer (Optima 2000DV, Perkin Elmer, USA).

### Statistical analysis

All experiments were carried out three times with three biological replicates each. Data for the each experiment was subjected to analysis of variance (ANOVA) to determine differences and expressed as the mean ± SE (standard error of the mean). Statistical significance was determined at *P ≤ 0*.*05* and mean values that were significantly different within a treatment are indicated by similar letters. Total lipid composition was statistically analyzed by principal component analysis (PCA) and a heat map was generated.

## Results

### Seed germination assay

**Fig 1:**
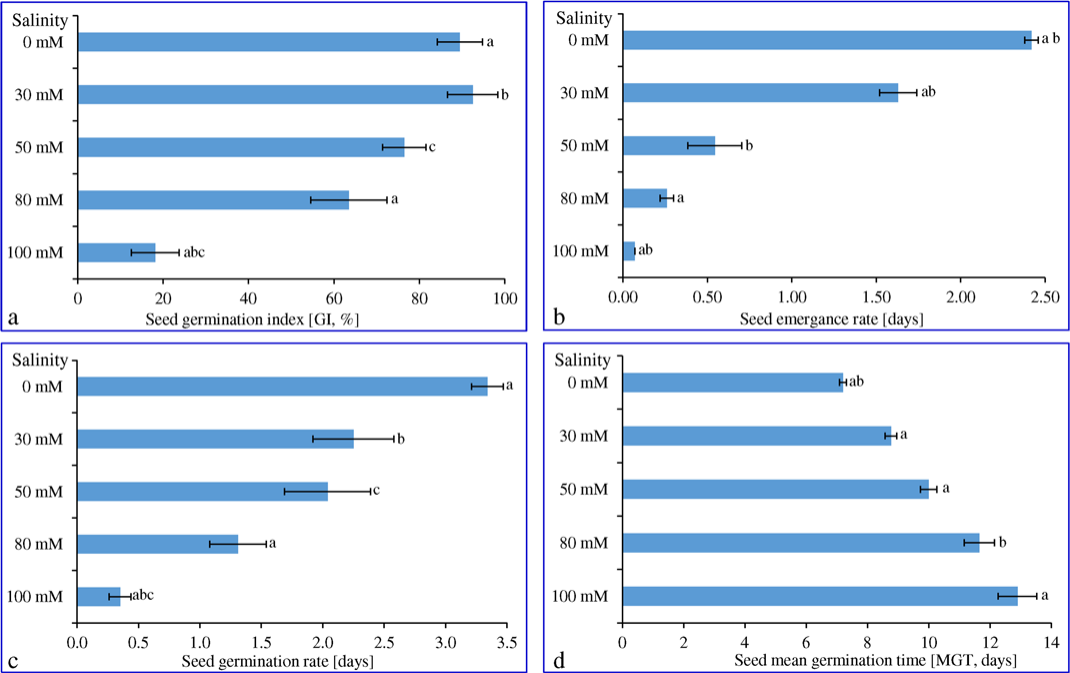
Seed germination assay of cumin under different salinity. Effect of different concentrations of NaCl on (a) seed germination index, (b) rate of seed emergence, (c) rate of seed and (d) mean germination time. Means ± SE followed by similar letters are significantly different at *P*<0.05.

Effect of different concentration of NaCl (0, 30, 50, 80 and 100 mM) on seed germination index (GI) of cumin was evaluated and delayed germination was observed under salinity. The highest germination index was recorded at 30 mM NaCl (92.5 ± 5.95), which decreased concomitantly with the further increase of NaCl concentration and reached lowest (18.15 ± 5.58) at 100 mM NaCl (Fig 1). Similarly, rate of seed emergence and germination were decreased in tandem with varying NaCl concentration (Fig 1). Seed emergence rate decreased radically (*P*<0.05) about 1.5, 4.5, 9 and 34-fold at 30, 50, 80 and 100 mM NaCl concentration, respectively compared to control condition (0 mM NaCl). The decrease in seed germination rate at 30 and 50 mM NaCl concentrations was non-significant compared to control (0 mM NaCl), whereas a significant decrease (*P*<0.05) was observed at 80 and 100 mM NaCl. About 2.5 and 9.5-fold decrease in germination rate was found at 80 and100 mM NaCl respectively.

Salinity had a significant effect on the mean germination time (MGT) and seeds with low MGT would germinate faster. In contrast to rate of emergence, germination and GI; mean germination time (MGT) was increased significantly (*P*<0.05) along with the salinity (Fig 1). The lowest mean average time (about 7 days) was observed in control (0 mM NaCl), which increased concomitantly and reached maximum about 13 days in 100 mM NaCl. About 9, 10 and 12 days were recorded as MGT for 30, 50 and 80 mM NaCl concentrations respectively.

### Morphological analysis

Salt stress-induced impairments were observed on growth of cumin plants, grown under different NaCl (0, 30, 50, 80 and 100 mM) concentrations (S1 Fig). Plant biomass decreased concomitantly with the increase of NaCl concentrations and a significant difference (*P<0*.*05*) was observed in fresh biomass (Fw) of seedlings at all treatments (S1 Fig). The maximum fresh weight was observed at 0 mM NaCl, which decreased about 23, 45, 67 and 91% in 30, 50, 80 and 100 mM NaCl concentrations, respectively. Salinity also influenced the dry weight of seedlings at all measured NaCl concentrations. There was a significant decrease (*P<0*.*05*) of 23, 37, 49 and 84.5% dry weight of seedlings, grown at 30, 50, 80 and 100 mM NaCl, respectively compared to 0 mM NaCl concentration (S1 Fig).

Salinity also influenced the length of root and shoot of cumin seedlings. Root length decreased significantly (P<0.05) along with salinity stress treatments (S1 Fig). At 30 mM NaCl, root length was reduced 22%, similarly 43 and 69% reduction were observed at 50 and 80 mM NaCl, whereas highest reduction about 90% was found in 100 mM NaCl compared to control plants, grown at 0 mM NaCl. Similarly, reduced shoot length was observed with increasing salt concentrations (S1 Fig). Shoot growth was decreased up to 20 and 34% at moderate salt concentrations, 30 and 50 mM NaCl respectively, whereas a significant reduction (*P<0*.*05*) of 74 and 90% were observed at 80 and 100 mM NaCl compared to seedling, grown at control condition. Apart from growth, salinity also influenced the root morphology of seedlings, grown at different NaCl concentrations (S2 Fig). Root hair were decreased gradually with increasing NaCl concentration, which completely absent at 100 mM and short radicles were emerged.

### Chlorophyll content

There was no significant difference in the contents of photosynthetic pigments (Chl a, Chl b and carotenoid) between plants, grown at control condition (0 mM) and 50 mM NaCl, whereas high salinity (100 mM NaCl) caused a significant reduction (*P<0*.*05*) and alternations in photosynthetic pigments (S3A Fig). Cumin plants showed an approximately 17 and 50% reduction of chlorophyll a and b, respectively under 100 mM NaCl compared to control condition. Plants grown at high salinity (100 mM) showed 28 and 25% reduction in total chlorophyll (Chl a + Chl b) and carotenoid contents, respectively.

### Amino acid, total soluble sugar, phenolic and flavonoid contents

In total, 15 amino acids were detected and quantified using HPLC (S3B Fig). The amount of amino acids (except asparagine) increased concomitantly with salt stress and elevated amount was detected under stress compared to the control condition. Glycine and leucine were major amino acids followed by proline and glutamate under control conditions (0 mM NaCl), whereas an abrupt rise in leucine, glycine and proline contents were observed at both 50 mM and 100 mM of NaCl concentrations. Leucine content increased about 2-fold in high NaCl concentration, similarly elevated contents of isoleucine, glycine and proline were also observed under high salinity compared to control condition (0 mM NaCl).

About 130 mg total sugar was detected per gram fresh weight of plant sample. A slight decrease was observed in the total sugar content (about 115 mg per FW) under 50 mM salt stress, which increased to about 140 mg per FW under 100 mM salt stress compared to control condition (S3C Fig). Similarly, total phenolic and flavonoid contents were decreased initially to some extent (about 1.4-fold at 50 mM), thereafter increased (about 1.2-fold at 100 mM) under salt stress condition (S3D Fig).

### Total antioxidant and DPPH inhibition activity

Total antioxidant activity of cumin plant was analyzed under stress condition and expressed in the terms of % inhibition of decolorization of ABTS^.+^. Total antioxidant activity increased concurrently with the extract concentration under all stress treatments (S4A Fig). At low concentration, the antioxidant activity of extract was almost similar under control and high stress (100 mM) condition but at higher concentration activity was reduced. It was observed that the scavenging of DPPH radicals was not affected by stress conditions (S4B Fig).

### H_2_O_2,_ Proline and MDA contents

**Fig 2:**
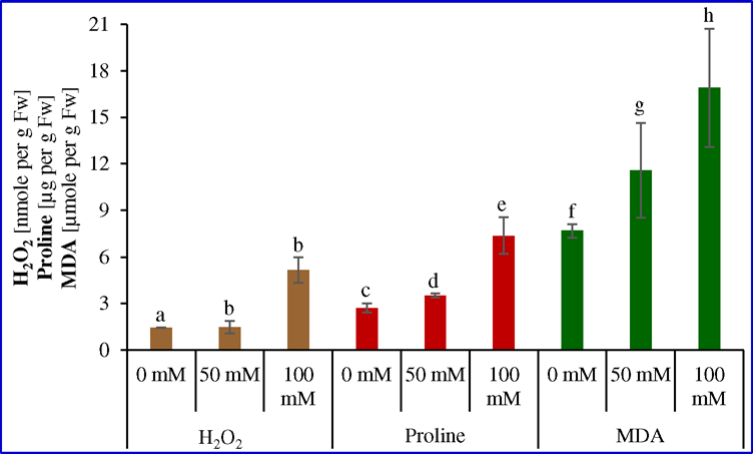
H_2_O_2,_ Proline and MDA contents of cumin seedling under varying salinity stress. Means ± SE followed by similar letters are significantly different at *P*<0.05.

H_2_O_2,_ proline and MDA contents were increased concomitantly with stress treatments (Fig 2). H_2_O_2_ and proline contents were increased by approximately 3-fold and 2.7-fold under 100 mM salinity stress compared to control plants. Lipid peroxidation was compared by estimating the MDA content (which is produced after lipid peroxidation that accumulated in leaves) under stress conditions. The MDA content was increased about 1.5- and 2.2-fold under stress treatment (50 and 100 mM, respectively) compared to the control conditions.

### Relative water content and Electrolyte leakage

**Fig 3:**
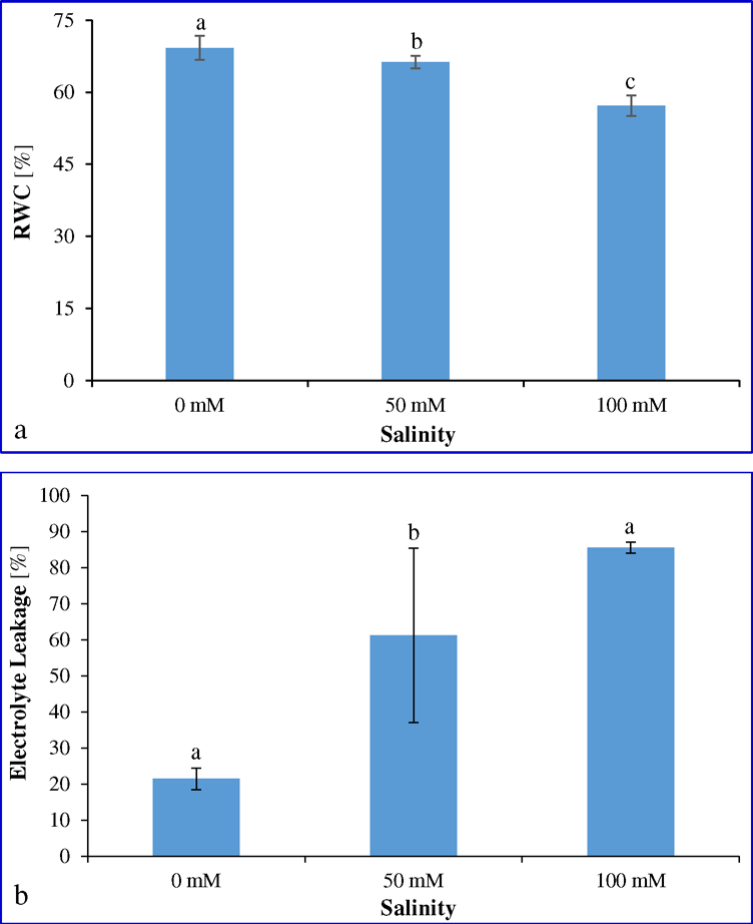
Physiological analysis of cumin under different salinity. Effect of different NaCl concentrations on (a) Relative water content and (b) Electrolyte leakage. Means ± SE followed by similar letters are significantly different at *P*<0.05.

Water retention ability of cumin leaves was evaluated under stress-treatment conditions (Fig 3). Relative water content (RWC) remained unchanged up to 50 mM NaCl stress, thereafter decreased significantly at 100 mM NaCl. Electrolyte leakage from cells due to membrane damage was assessed during salinity stress (Fig 3). About 2.8-fold electrolyte leakage was found in 50 mM, which increased further 4-fold at 100 mM NaCl stress compared to control condition.

### Enzyme activity

**Fig 4:**
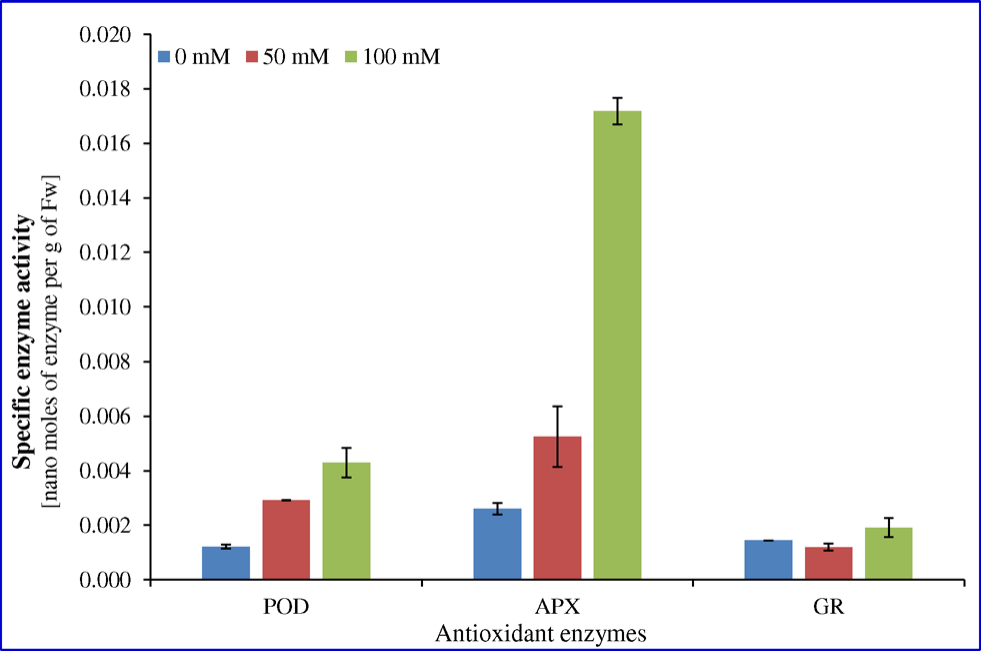
Enzyme activity under different salinity. Specific enzyme activity of POD, APX and GR in cumin seedlings under salinity stress. Value represents the mean ± SE.

Specific activity of enzymes was determined under varying salt stress treatments and represented as nano moles of enzymes present per gram of protein per gram of fresh weight sample (Fig 4). Specific activity of enzymes, peroxidase (POD) and ascorbate peroxidase (APX) increased concomitantly with salinity (0–100 mM NaCl). However, specific activity of glutathione reductase (GR) decreased at 50 mM NaCl stress, thereafter increased slightly under 100 mM NaCl stress compared to control condition.

### Ion contents

Different mineral ions (Ca^2+^, Mg^2+^, K^+^ and Na^+^) were determined under varying NaCl concentrations (S5 Fig). In cumin seedlings, Ca^2+^ and Mg^2+^ contents were decreased (in-significantly) in root and shoot at high salinity (50 and 100 mM NaCl). An elevated K^+^ content was found in root, however lower content was observed in shoot at 100 mM NaCl. In contrary, K^+^ content decreased in root and increased in shoot at 50 mM NaCl compared to control condition (0 mM NaCl). A concomitant decrease of Na^+^ content was observed in root, however increase progressively in shoot with NaCl concentration.

### Fatty acid profiling

**Fig 5:**
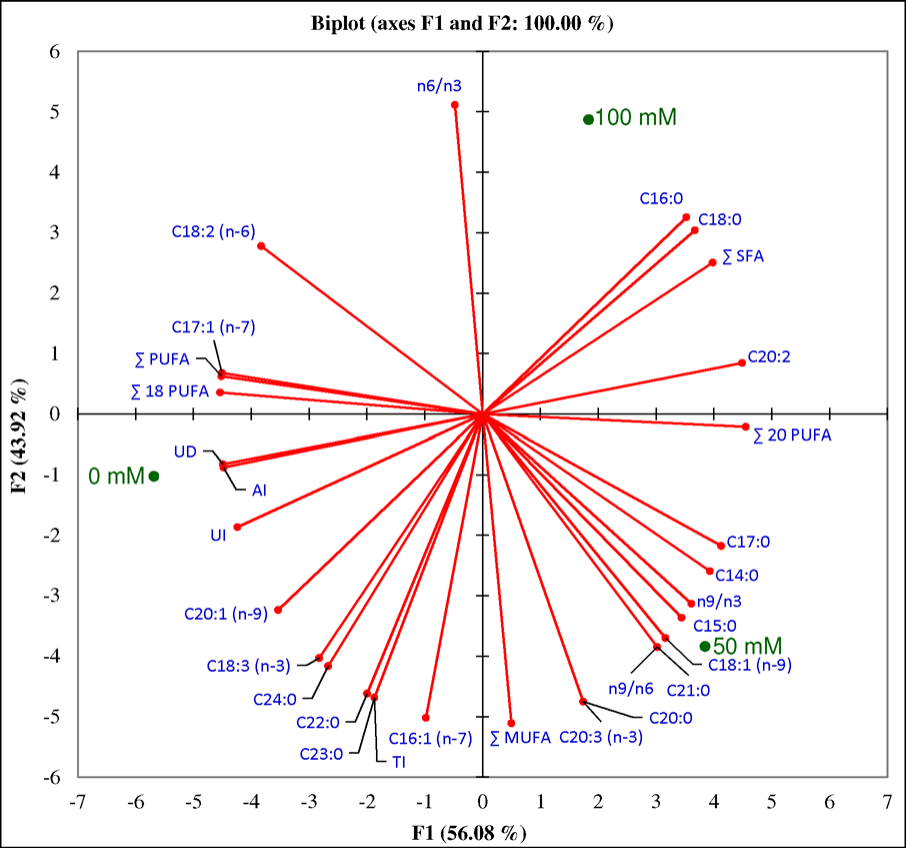
Bi-plot of total lipid and fatty acid composition. Plot was obtained PCA analysis of FA groups with first two principal components.

**Fig 6:**
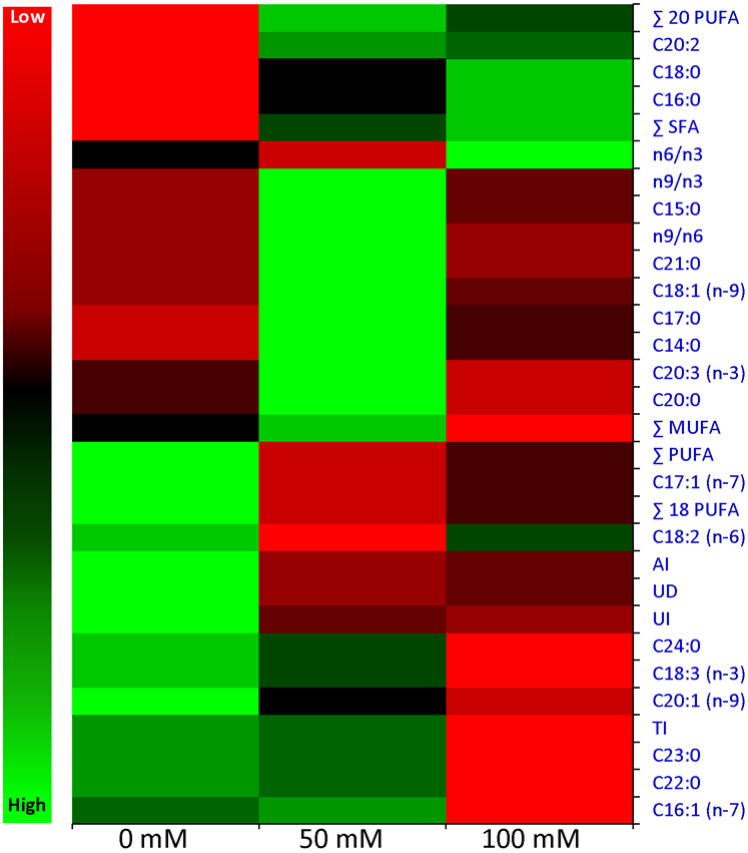
Heat map of total lipid and fatty acid composition. Differential expression of lipid and FA composition under salinity stress.

Total fatty acid (FA) content of cumin shoot was comprised of a range of FAs (C14 to C24), including 53% polyunsaturated, 39% saturated and 8% monounsaturated fatty acids (Table 1). FA composition was dominated with linolenic acid (C18:2; 34%) followed by palmitic acid (C16:0; 27%), alpha-linoleic (C18:3; 19%), stearic acid (C18:0; 9%) and palmitoleic acids (C16:1; 7%). Under salinity stress, a change in the percent quantity of FAs was observed (Table 1). Quantities of myristic acid (C14:0), pentadeconoic acid (C15:0), heptadecanoic acid (C17:0), oleic acid (C18:1), cis-11,14,17-eicosadienoic acid (C20:3) and heneicosanoic acid (C21:0) were increased with salinity up to 50 mM NaCl, thereafter declined slightly on further increase of NaCl concentration (100 mM) but percent quantity remained higher than the control condition. Elevated quantity of palmitoleic acid (C16:1) was detected at 50 mM NaCl, which decreased suddenly at 100 mM NaCl and reached lower than the quantity of control condition (0 mM NaCl). Synthesis of palmitic acid (C16:0) and stearic acid (C18:0) increased concomitantly with salinity, in contrast quantities of alpha-linolenic acid (C18:3), cis-11-eicosadienoic acid (C20:1), behenic acid (C22:0), tricosanoic acid (C23:0) and lignoceric acid (C24:0) decreased concurrently with salinity. Cis-10-heptadecanoic acid (C17:1) was detected under control and high salinity (100 mM NaCl), whereas cis-11,14-eicosadienoic acid (C20:2) was synthesized under salt stress only. Quantity of linoleic acid (C18:2) decreased with salt stress, thereafter an elevated quantity was detected under high salinity (but lower than the control condition). Biosynthesis of arachidic acid (C20:0) remained unaffected under stress treatments. Overall, amount of saturated FAs increased gradually with salinity, whereas unsaturation index and degree of unsaturation changed arbitrarily along with the percent quantity of unsaturated FAs (MUFA and PUFA). Principal component analysis (PCA) indicated that total lipid and fatty acid composition was significantly influenced by salinity stress (Fig 5) and heat map showed that fatty acid composition changed under NaCl stress (Fig 6).

**Table 1:** Effect of varying salinity on Fatty acid composition of cumin shoots.

Fatty Acid	Name of Fatty acid	0 mM	50 mM	100 mM
*Fatty acid composition*				
C14:0	Myristic acid	0.31±0.02	0.53±0.13	0.38±0.01
C15:0	Pentadecanoic acid	0.18±0.01	0.25±0.07	0.19±0.01
C16:0	Palmitic acid	26.98±1.33	27.98±1.38	28.85±0.32
C16:1 (n-7)	Palmitoleic acid	6.72±0.17	6.86±0.32	5.64±0.03
C17:0	Heptadecanoic acid	0.17±0.01	0.22±0.05	0.19±0.01
C17:1 (n-7)	Cis-10-Heptadecanoic acid	0.03±0.01	—-	0.01±0.01
C18:0	Stearic acid	9.06±1.2	10.28±1.12	11.16±0.18
C18:1 (n-9)	Oleic acid	1.12±0.22	1.95±0.04	1.16±0.08
C18:2 (n-6)	Linoleic acid (LA)	34.30±1.4	31.27±0.17	33.45±0.18
C18:3 (n-3)	alpha-Linolenic acid (ALA)	18.62±0.85	18±1.29	16.83±0.24
C20:0	Arachidic acid (AA)	0.21±0.02	0.23±0.04	0.2±0.01
C20:1 (n-9)	Cis-11-Eicosenoic acid	0.28±0.05	0.21±0.03	0.15±0.02
C20:2	Cis-11,14-Eicosadienoic acid	—-	0.24±0.06	0.23±0.01
C20:3 (n-3)	Cis-11,14,17-Eicosadienoic acid	0.16±0.01	0.2±0.09	0.14±0.01
C21:0	Heneicosanoic acid	0.10±0.01	0.12±0.04	0.1±0.01
C22:0	Behenic acid	0.81±0.09	0.78±0.1	0.6±0.01
C23:0	Tricosanoic acid	0.22±0.02	0.21±0.05	0.13±0.01
C24:0	Lignoceric acid	0.72±0.04	0.68±0.09	0.59±0.04
*Lipid composition*				
∑ SFA	Saturated fatty acids	38.77±3.56	41.27±2.89	42.39±0.79
∑ MUFA	Monounsaturated fatty acids	8.15±1.15	9.02±0.66	6.96±0.19
∑ PUFA	Polyunsaturated fatty acids	53.08±1.02	49.47±0.57	50.64±0.19
∑ 18 PUFA	—-	52.92±1.02	49.26±0.48	50.27±0.16
∑ 20 PUFA	—-	0.16±1.22	0.44±0.82	0.37±0.24
U.I	Unsaturation index	133.1±4.21	126.63±3.93	125.21±0.96
U.D	Degree of unsaturation	114.32±3.36	107.96±2.43	108.25±0.71
n6/n3	—-	1.83±1.24	1.73±1.09	1.97±0.28
n9/n3	—-	0.07±1.22	0.12±0.98	0.08±0.26
n9/n6	—-	0.04±1.22	0.07±0.97	0.04±0.26
AI	Atherogenic index	44.37±0.97	42.59±0.36	42.65±0.15
TI	Thrombogenic index	84.72±0.81	84.34±2.03	81.30±0.31

Fatty acid composition of cumin under different concentration of NaCl stress given in means ± SE as % of total FAMEs

### Metabolite profiling

A total of 45 differentially expressed metabolites were identified in cumin plants grown under varying salinity (0, 50 and 100 mM NaCl) using liquid chromatography–TOF–mass spectrometry (LC-TOF-MS; Table 2). Metabolites methylhexacosane or dimethylpentacosane (m/z 381.44; dppm 14), a plant origin nutritive acyclic hydrocarbons and a sex pheromone component respectively, were constitutively synthesized under control and stress condition, however about 15 metabolites were expressed in control condition only. Most of these metabolites were flavonoid and contain antioxidant activities. A metabolite docetaxel (m/z 824.33; dppm 22) possesses anticancerous activity, whereas metabolite megalomicin (m/z 975.61; dppm 10) owns antiparasitic, antiviral and antibacterial properties. Synthesis of three metabolites, including a phospholipids (m/z 977.5; dppm 16), an intermediate of phenylalanine metabolism (m/z 313.04; dppm 2; 2-Hydroxy-6-ketononatrienedioate) and/ or 3-(2-Carboxyethenyl)-cis,cis-muconate (m/z 313.04; dppm 2), a plant origin flavonoids (flavan-3-ol) with anti-oxidant activity were observed in control and moderate salinity (50 mM NaCl) only, and not detected under 100 mM NaCl stress condition. Remarkably, three important metabolites were expressed under control and high salinity stress condition, and these include two natural flavonoid (flavonol); kaempferol (m/z 755.21; dppm 9) and quercetin (m/z 961.27; dppm 8), and an O-methylated anthocyanidin (a primary plant pigment; m/z 475.64; dppm 4) peonidin. A total of 22 metabolites were detected under high salinity (100 mM) only, of which 12 metabolites were associated with fatty acids metabolism (either short chain FA, intermediate or enzymes) and remaining were alkaloids or flavonoids.

**Table 2:** Probable metabolites and their possible application/ role identified in cumin under salinity stress.

Probable Metabolites	0 mM	50 mM	100 mM	Possible Properties/ Function/ Applications
Methylhexacosane	**√**	**√**	**√**	Plant origin nutritive acyclic alkanes (acyclic hydrocarbons)
Dimethylpentacosane	**√**	**√**	**√**	A sex pheromone component
Small peptides [Asn + Asn + Gly + Asn]	**√**	nd	nd	Small peptide
Sulfoacetyl-CoA	**√**	nd	nd	Enzyme involeved in NADP redox reaction
CDP-DG(16:0/18:0) or CDP-DG(18:0/16:0)	**√**	nd	nd	Involved in amino sugar and nucleotide sugar metabolism
Salvianolic acid L	**√**	nd	nd	A phenolic compound with antioxidant activities
Luteolin	**√**	nd	nd	A flavone (a type of flavonoid) compound
Syringetin	**√**	nd	nd	An O-methylated flavonol (a type of flavonoid)
Petunidin	**√**	nd	nd	An O-methylated anthocyanidin (a natural organic compound)
Isorhamnetin	**√**	nd	nd	An O-methylated flavonol (a type of flavonoid)
Sexangularetin	**√**	nd	nd	A plant origin flavonoid
Glycan:Galb1-3[Neu5Aca2,6]GalNAca-Thr	**√**	nd	nd	A glycan
Jionoside B1	**√**	nd	nd	Antioxidant
Precorrin 1	**√**	nd	nd	An intermediate of vitamin B12 biosynthesis
Docetaxel M2	**√**	nd	nd	An anticancerous compound
Megalomicin C2	**√**	nd	nd	A macrolides and lactone polyketides with antiparasitic, antiviral and antibacterial properties
Sphingolipids [SP]: cer(d18:1/24:1(15Z))	**√**	nd	nd	Sphingolipids
Phosphatidylinositol phosphate	**√**	**√**	nd	Involved in biosynthesis of secondary metabolites
2-Hydroxy-6-ketononatrienedioate	**√**	**√**	nd	An intermediate of phenylalanine metabolism
3-(2-Carboxyethenyl)-cis,cis-muconate	**√**	**√**	nd	A plant origin flavonoids (flavan-3-ol)
Kaempferol	**√**	nd	**√**	A natural flavonol (flavonoid)
Quercetin	**√**	nd	**√**	A natural flavonol (flavonoid)
Peonidin	**√**	nd	**√**	An O-methylated anthocyanidin (a primary plant pigment)
Carthamin	nd	nd	**√**	A natural plant pigment used as a food additive (dye and a food coloring agent)
Salvianin	nd	nd	**√**	A polyacylated anthocyanin
Cyanidin	nd	nd	**√**	A natural organic compound (a type of anthocyanidin)
2-Hydroxy-3-chloropenta-2,4-dienoate	nd	nd	**√**	An intermediate in fatty acid metabolism
Prenol Lipids [PR]: Epoxy isoprenoid	nd	nd	**√**	A plant origin isoprene (branched-chain unsaturated hydrocarbon)
7-Pentacosanone	nd	nd	**√**	Aliphatic Acyclic Compounds
Glycerophospholipids: PA(O-16:0/15:1(9Z))	nd	nd	**√**	Lipid
Glycerophospholipids: PG(12:0/13:0)	nd	nd	**√**	Polar Lipid
Linoleamide	nd	nd	**√**	An unsaturated analog of endogenous sleep-inducing lipid
Coixenolide	nd	nd	**√**	FA (fatty acid)
5,9-tetracosadienoic acid	nd	nd	**√**	FA (fatty acid)
3-Epidemissidine	nd	nd	**√**	A plant origin alkaloid
YGM 5B	nd	nd	**√**	Flavonoid
N1-(5-Phospho-D-ribosyl)-ATP	nd	nd	**√**	Involve in amino acid (histidine) metabolism
Sphingolipids [SP]: Cer(d18:1/26:1(17Z))	nd	nd	**√**	Lipid
Nonanoyl-CoA	nd	nd	**√**	A medium-chain fatty acyl-CoA involve in fatty acid metabolism
Octanoyl-CoA	nd	nd	**√**	Involve in FA degradation (beta oxidation) in peroxisomes
2,6-Dihydroxycyclohexane-1-carboxyl-CoA	nd	nd	**√**	Enzyme involve in FA metabolism
6-Carboxyhexanoyl-CoA (Pimeloyl-CoA)	nd	nd	**√**	Enzyme involve in FA metabolism
Tetradecanoyl-CoA	nd	nd	**√**	Enzyme involve in FA metabolism
Spinacetin	nd	nd	**√**	An O-methylated flavonol
Mesembryanthin	nd	nd	**√**	Flavonoid O-Glycosides

nd: not detected and √: present (detected)

## Discussion

Cumin is an important medicinal and spice crop cultivated in the arid and semi-arid regions of the world, where saline soil is widely distributed, unsuitable for agriculture [12]. Plant is good source of minerals like iron, copper, zinc and manganese and B-complex vitamins such as thiamine, niacin, riboflavin and antioxidant vitamins like A, E and C [47]. Cumin and value added products of cumin seeds have gained importance in functional food with wide range of health benefits, dietary supplements, cosmetics and other nutraceutical applications. It was observed that biomass of plant, plant height and physiological factors like germination percentage and stage of germination were significantly affected under the influence of different levels of salt (Fig 1). Seed germination process was delayed under salinity, resulting in the decrease of germination index with increasing salinity stress (Fig 1). Water uptake is very crucial for seed germination process, however presence of NaCl in the surrounding medium creates negative water potential resulting in retardation of water imbibition by seeds [48]. As a consequence, various metabolic perturbations are induced by salinity stress that comprise of slow or less mobilization of reserve nutrients, arrest in cell division, decreased synthesis of nucleic acid and thus causing injury to germinating embryos [49]. This delayed the time of radicle emergence and hence increases time needed for germination thus affecting the seed vigor. The reason for increased lag period could be attributed to the osmotic effect or accumulation of specific ions up to a toxic level [48]. This observation was based on a report that deteriorated seeds require more time for metabolic repair before the process of seed germination could begin [50].

The energy production of plant system is dependent on the photosynthetic ability to synthesize various metabolites that lead to accumulation of biomass and promote growth. Under high saline conditions, rate of photosynthesis gets highly affected due to non-availability of raw materials required for photosynthesis, such as uptake of essential nutrients and decrease in water absorption from roots [51]. Reduced fresh biomass accumulation was observed in cumin seedlings, which might be due to inhibition of cell elongation (S1 Fig). Higher salinity have also been reported to induce changes in protein structure, increase in cytoplasmic RNAase activity, leading to decrease in DNA synthesis, creating many cellular menaces to activity required for development processes in plants [52].

Photosynthesis, the most essential physiological process, is involved in energy metabolism of all plant system. This includes numerous different types of pigments, such as chlorophyll (a, b and a+b) and carotenoid. They are involved in synthesis of metabolites that promote the plant growth and development. However increase in soil salinity can cause abrupt changes in various photosynthetic pigments [51]. Thus chlorophyll content can be studied as one of the markers of cellular stress, and its decreased level can provide the evidence of severity of stress in plants [53]. In cumin leaves, Chl a, Chl b, total Chl a+b and carotenoid contents remain unaffected at 50 mM NaCl in comparison to 0 mM NaCl (S3A Fig). However at higher salinity (100 mM NaCl), a decrease of 17, 50 and 28% was observed in Chl a, Chl b and Chl (a + b) respectively (S3A Fig). Results obtained in this study were in agreement with those of Shobbar *et al*. [54] for rice. On the onset of stress, ROS scavengers cannot efficiently deal with the higher rate of ROS generation, which in turn inhibits the PSII repair system and synthesis of D1 proteins in chloroplasts [55]. Carotenoids are accessory light harvesting pigments, preventing the photosynthetic pigments from photo-damage. They are reported to stabilize the phospholipids present in thylakoid and scavenge various reactive oxygen species generated during stressful environment of salinity. The carotenoid content was decreased by 25% in cumin leaves at 100 mM NaCl stress as compared to 0 mM NaCl (S3A Fig).

Plants are known to synthesize soluble sugars under salinity stress. Soluble sugars are involved in biosynthetic process and balance the osmotic strength of cytosol with that of vacuole [56]. The increased accumulation of total sugar at higher NaCl (100 mM) concentration defines its role as an osmoprotectant in cumin seedlings, which are subjected to salinity stress with respect to 0 mM NaCl (S3C Fig). Thus increase of sugar content may be the direct consequence of either inhibition of starch biosynthesis or degradation of starch due to Na^+^ ion toxicity [57]. Present study revealed that various essential and non-essential amino acids are accumulated in cumin seedlings under salinity stress (S3B Fig). The result signifies that cumin is a rich source of amino acids and can act as dietary supplement due to nutritional benefits [58]. Essential amino acids, such as leucine and isoleucine were found to accumulate more under different level of salinity. The elevated amount of nonessential amino acids, such as glycine and proline were also observed along with glutamate under stress. Amino acids, especially proline, was synthesized under stress condition to provide osmotic protection to the plant. Previously, it was found that the amino acid content increased in *Aloe vera* [59] and *Salicornia brachiata* [14] during salinity stress at seedling stage. The increased level of free amino acids in the cell cytoplasm may play an important role in osmotic adjustments, which are also involved in the stability and integrity of cellular membranes in saline environment [14].

The enhanced production of ROS is the result of imbalance created in photo-oxidative process occurring in the plant cell. Several abiotic stresses, such as salinity and drought are known to cause a significant increase in H_2_O_2_ that causes lipid peroxidation of cellular membranes [60]. Exposure of cumin seedlings to 100 mM salt treatment for 7 day caused a significant increase in the level of H_2_O_2_ (3.6-fold) with respect to 0 mM NaCl (Fig 2). During abiotic stress, reduced activity of photosystem II generates free radicals that ultimately produce H_2_O_2_ by Mehler reaction, occurring in the chloroplast and mitochondria [56]. The elevated level of H_2_O_2_ in the plant extract of cumin attributed the lethal effect of higher salt concentration on biochemical reactions within the cell. Proline is an important amino acid with a wide range of biological functions in plants and known for the maintenance of osmotic adjustments and membrane stability [61]. In present study, significant increase in proline content in cumin under different salinity stress reflected that NaCl has resulted in osmotic stress, so plant synthesized proline as an osmoprotectant (Fig 2). A significant rise in proline content was observed at 100 mM NaCl which was 2.7-fold higher than 0 mM NaCl. The proline accumulation in cell increased in concentration dependent manner with respect to different NaCl concentration. The stress protective function of proline have been illustrated in different plant species [62–66].

The level of salt tolerance in crop plants can also be assessed physiologically by measuring change in relative water content and electrolyte leakage (Fig 3). Results are in agreement with previous studies conducted in different plant species, in which marginal or severe reduction in water status were reported due to stress arising from saline environment [56]. Loss of the membrane integrity and stability is a common symptom developed in plants under salinity stress [67]. This condition arises due to formation of free radicals and lipid peroxidation. Present study found a significant increase in the MDA content at both moderate (50 mM) and high (100 mM) salt stress (Fig 2). Level of lipid peroxidation was increased by 1.5-fold and 2.2-fold with increase in salt concentration from 50 to 100 mM NaCl, respectively as compared to 0 mM NaCl. The study is supported by previous findings; rice [54] and groundnut [65, 68], in which MDA content increased significantly under salt stress. So cumin plant is sensitive to different level of salinity and undergoes severe membrane disruption.

Abiotic stress promotes the synthesis of various secondary metabolites possessing antioxidant activity. Phenolic and flavonoid contents are generally accumulated in response to salinity stress [69]. Phenolics and polyphenolics are ubiquitous in foods, beverages and plants including spices, and are an essential part of the human diet [70]. Being a spice crop of semi-arid regions, cumin is known to possess an advanced antioxidant system, needed to combat the dry and stressful growing environment [58]. Plant extracts show enhanced total antioxidant activity and total flavonoid content under stress conditions (S3D and S4A Figs). In contrast, a decrease in total phenolic content was found under stress in cumin leaves (S3D Fig). Antioxidant activities and phenolic compounds present in cumin seeds varied, depending on their geographical origin and solvents used for extraction [47, 58]. Antioxidants play a key role in health promotion by inhibiting oxidation processes of functional food ingredients and its activity can be assayed with different mechanisms [71]. The promising antioxidant activity, possessed by plant products could be due to the abundance of phenolic compounds viz. flavonoids, phenolic acids and polyphenols etc. [14, 70]. Synthesis of these compounds is reported to increase significantly under abiotic stress, known to preclude oxidative modification of lipoproteins and lipid peroxidation of membranes of the plant cell [47, 56]. The antioxidant activity measured by 1, 1- diphenyl 2- picrylhyorazyl (DPPH) system was found to be the highest in the extract of 0 mM NaCl salt. In salinity treatment, scavenging activity was lower at both 50 and 100 mM NaCl (S4B Fig). This might be the result of in-ability of scavenging system to detoxify reactive oxygen species generated under salt treatment. Salinity has been reported to impair the DPPH radical scavenging activity in many crops. DPPH activity was decreased by 35% due to salinity stress in *Gossypium hirsutum* [72]. Also in coriander fruit, the antioxidant activity was highly suppressed under saline conditions leading to increased sensitivity towards salinity stress [73]. These activities may be dependent on the concentration of phenolic compounds since in cumin leaves, total phenolic content decreased under salinity stress and hence controls the efficiency to detoxify free radicals [58]. Previous findings have shown a direct correlation between the antioxidant activity and total phenolic amounts of many plants, including coriander and Salicornia [14, 73]. Furthermore, it was well established that salinity-induced oxidative stress could be mitigated through the overexpression of antioxidant enzymes. In the present study, specific activity of antioxidant enzymes (peroxidase, POD; ascorbate peroxidase, APX and glutathione reductase, GR) were increased with salinity (Fig 4). In earlier studies, it was found that antioxidant enzymes, such as POD, APX and GR play a vital roles to provide salt endurance to the plant. Antioxidant enzymes APX and GR are key players in the ascorbate-glutathione cycle by reducing H_2_O_2_ to water through oxidizing ascorbate to monodehydroascorbate [74].

It is well known that plant cell membrane is dynamic in nature and lipid composition depends on surrounding environment. Essential fatty acids (FAs) cannot be naturally made in a sufficient quantity therefore outsourced by the human body from food [75]. Furthermore, PUFAs are considered as an important nutritional indicator and plants that contain it, especially linoleic acid, are considered to have medical importance. Present study showed that cumin shoot contains 53% PUFAs and thus reveals medicinal importance of the plant (Table 1). Two essential FAs, linoleic acid (C18:2) and alpha-linolenic acid (C18:3), were major FA in cumin shoots contributing 34% and 19% to total FAs, respectively. Linoleic acid is reported as major FA which quantitatively decreased under drought stress [47, 58]. Membrane fluidity is influenced by the degree of FA unsaturation and thus provides protection to the cell and maintained an appropriate environment for the functioning of membrane under abiotic stress [76]. In the present study, saturated FAs increased concomitantly with salinity, whereas unsaturation index and degree of unsaturation were changed arbitrarily (along with the percent quantity of unsaturated FAs), which may result in the variation of plasma membrane lipid composition, required for the membrane integrity. Present study was in the agreement with previous reports, in which unsaturated FAs decreased significantly in cumin shoots and seeds under drought conditions [47, 58] leading to the membrane deterioration and permeability. Principal component analysis (PCA) exhibited a statistical distinction of total lipid and fatty acid composition under salinity stress (Fig 5) and heat map (Fig 6). PCA explained 100% variations (PC1- 56.08% and PC2-43.92%) and revealed a correlation between FA biosynthesis and salinity stress.

Previous studies have also reveal that cumin seeds are excellent source of secondary metabolites, such as polyphenols with free radical scavenging properties and have wide applications in food industries [58]. Major secondary metabolites isolated from cumin seed are 1-O-β-D-glucopyranoside [77], β-D-glucopyranoside [78] and cuminoside A and B [77] etc. The metabolic activity of plant is highly affected by salinity induced nutritional imbalance or accumulation of specific ions up to a toxic level [53]. A correlation between plant secondary metabolism and defense response is extensively documented [79]. Alkaloids, anthocyanins, flavonoids, quinones, lignans, steroids and terpenoids are major plant secondary metabolites that are widely used for pharmaceutical applications [80]. Secondary metabolites of medicinal plants have a great value and no earlier studies were available regarding the metabolite profiling of cumin shoots, so far. Synthesis of metabolites are known to be environment dependent [14] therefore differential synthesis of metabolites were identified under varying salinity in cumin. In the present study, a total of 45 differentially expressed metabolites were identified from cumin shoots, grown under varying salinity along with their possible applications (Table 2). Most of the metabolites, including luteolin, salvianolic acid, kaempferol and quercetin are of phenolic, flavonoid or alkaloids in nature and contain antioxidant activities [5, 81–82]. These metabolites are known as nutritive supplements and thus provide functional value to the plant. Furthermore, a strong positive correlation was reported between antioxidant activity and total flavonoid content in *Gynura procumbens* [83]. A flavonoid quercetin (quercetin-3-O-α-L-rhamnopyranoside) obtained from *Toona sinensis* contained antioxidant with anticancer activities [84], whereas quercetin (quercetin 3-β-D-glucoside), isolated from *Satureja montana* was reported to be an antioxidant [85]. Despite of flavonoid, cumin shoots own metabolites having various pharmacological activities, such as antimicrobial, anti-daibetic, antiepileptic anti-infertility, anticancer, antioxidant and immunomodulatory [2, 4–5]. Metabolites with bioactivity such as anticancerous (docetaxel) and antimicrobial (megalomicin) were also identified along with intermediate metabolites involved in different pathways. Gagandeep *et al*. [86] demonstrated that cumin has an anticancer effect and inhibits the induction of gastric squamous cell carcinomas in mice.

## Conclusion

Cumin is an annual herb with numerous medicinal values. Present study showed that plant shoots are rich source of essential amino acids, phenolic compounds and fatty acids. Furthermore, the study evidenced that cumin shoots contain metabolites with bioactivities, which reveal medicinal potential of the plant. Study also provides useful insight about metabolic responses under salinity stress. In conclusion, nutritional antioxidants, scavenging activities, amino acids, alkaloids, flavonoids, essential FAs, PUFAs and metabolites make cumin shoots a promising functional food to be used as salad green with daily food as dietary supplement and also in nutraceutical industries.

## Supporting Information

S1 Fig Plant biomass under salt stress. Salt stress-induced impairments on plant biomass (fresh weight and fry weight) and length of root and shoots of cumin seedlings under salinity stress. Means ± SE followed by similar letters are significantly different at *P*<0.05.
(PPTX)

S2 Fig Root morphology under salt stress. Changes in root morphology of cumin seedling treated with (a) 0 mM, (b) 30 mM, (c) 50 mM, (d) 80 mM and (e) 100 mM of NaCl concentration.
(PPTX)

S3 Fig Chlorophyll, amino acid, soluble sugar, phenolic and flavonoid contents of cumin under salt stress. Chlorophyll content (S3a), amino acid (S3b), total soluble sugar (S3c), phenolic and flavonoid contents (S3d) of cumin seedling grown under salinity stress. Means ± SE followed by similar letters are significantly different at *P*<0.05.
(PPTX)

S4 Fig Total antioxidant and DPPH inhibition activity. Total antioxidant (S4a) and DPPH inhibition (S4b) activity of cumin seedling under varying salinity stress. Value represents the mean ± SE.
(PPT)

S5 Fig Determination of ions contents in cumin under salinity stress. Value represents the mean ± SE.
(PPTX)
