# Supplementary material for: Physio-Biochemical Composition and Untargeted Metabolomics of Cumin (Cuminum cyminum L.) Make It Promising Functional Food and Help in Mitigating Salinity Stress
Source: PLoS One. 2015 Dec 7;10(12):e0144469. doi: 10.1371/journal.pone.0144469 (PMC4671573; doi:10.1371/journal.pone.0144469)
Supplement: S1 Fig — Salt stress-induced impairments on plant biomass (fresh weight and fry weight) and length of root and shoots of cumin seedlings under salinity stress. Means ± SE followed by similar letters are significantly different at P<0.05. (PPTX) [file pone.0144469.s001.pptx]

## Slide 1
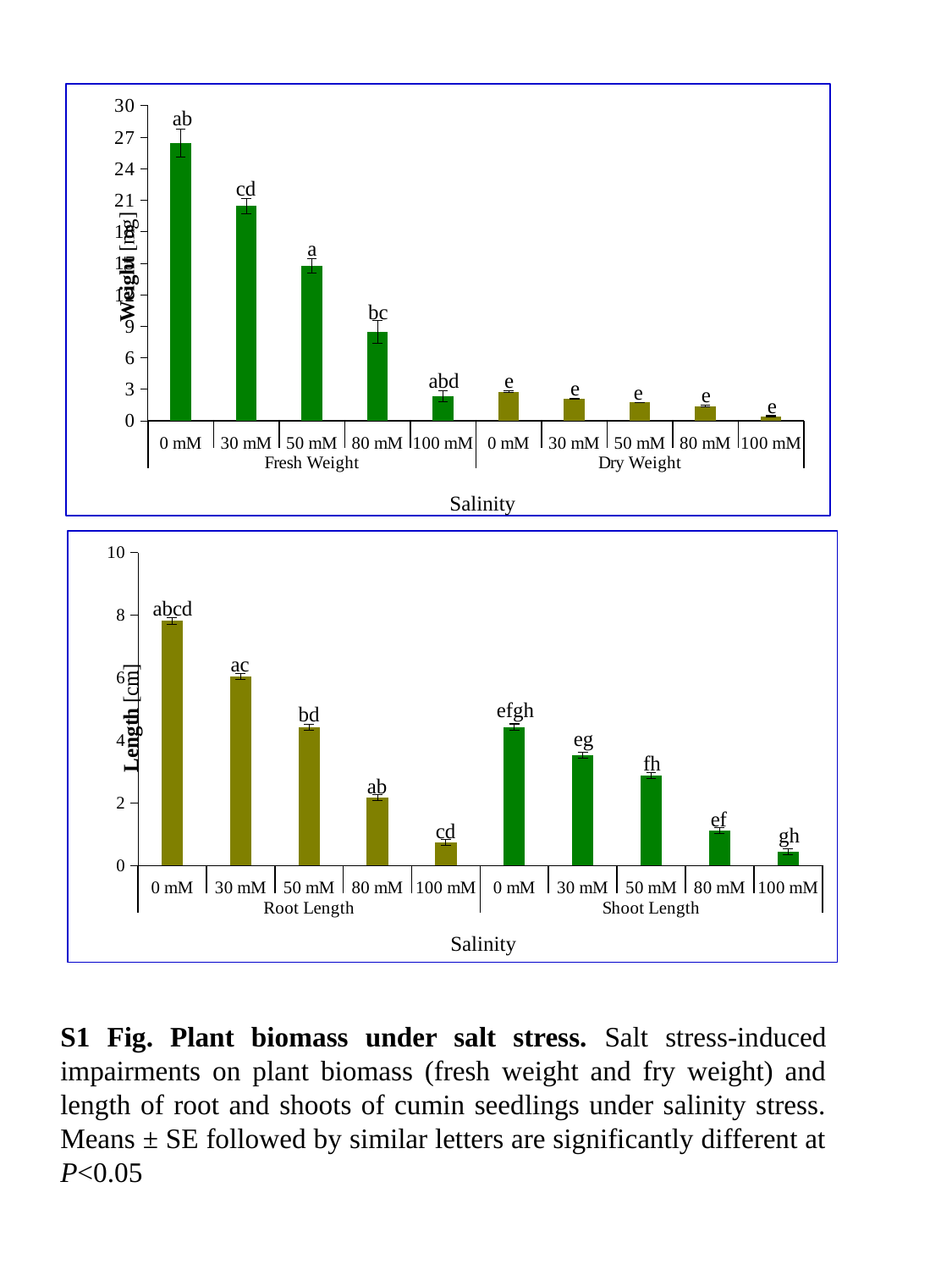

### Chart
| Category | Fresh Weight Dry Weight |
|---|---|
| 0 mM | 26.43 |
| 30 mM | 20.459999999999987 |
| 50 mM | 14.76 |
| 80 mM | 8.47 |
| 100 mM | 2.3499999999999988 |
| 0 mM | 2.7800000000000002 |
| 30 mM | 2.13 |
| 50 mM | 1.75 |
| 80 mM | 1.41 |
| 100 mM | 0.45 |ab
cd
a
bc
abd
e
e
e
e
e
### Chart
| Category | Root Length Shoot Length |
|---|---|
| 0 mM | 7.8199999999999985 |
| 30 mM | 6.04 |
| 50 mM | 4.42 |
| 80 mM | 2.17 |
| 100 mM | 0.7400000000000004 |
| 0 mM | 4.430000000000002 |
| 30 mM | 3.53 |
| 50 mM | 2.88 |
| 80 mM | 1.12 |
| 100 mM | 0.44 |abcd
ac
efgh
bd
eg
fh
ab
ef
cd
gh
Salinity
Salinity
S1 Fig. Plant biomass under salt stress. Salt stress-induced impairments on plant biomass (fresh weight and fry weight) and length of root and shoots of cumin seedlings under salinity stress. Means ± SE followed by similar letters are significantly different at P<0.05
