# Supplementary material for: Physio-Biochemical Composition and Untargeted Metabolomics of Cumin (Cuminum cyminum L.) Make It Promising Functional Food and Help in Mitigating Salinity Stress
Source: PLoS One. 2015 Dec 7;10(12):e0144469. doi: 10.1371/journal.pone.0144469 (PMC4671573; doi:10.1371/journal.pone.0144469)
Supplement: S2 Fig — Changes in root morphology of cumin seedling treated with (a) 0 mM, (b) 30 mM, (c) 50 mM, (d) 80 mM and (e) 100 mM of NaCl concentration. (PPTX) [file pone.0144469.s002.pptx]

## Slide 1
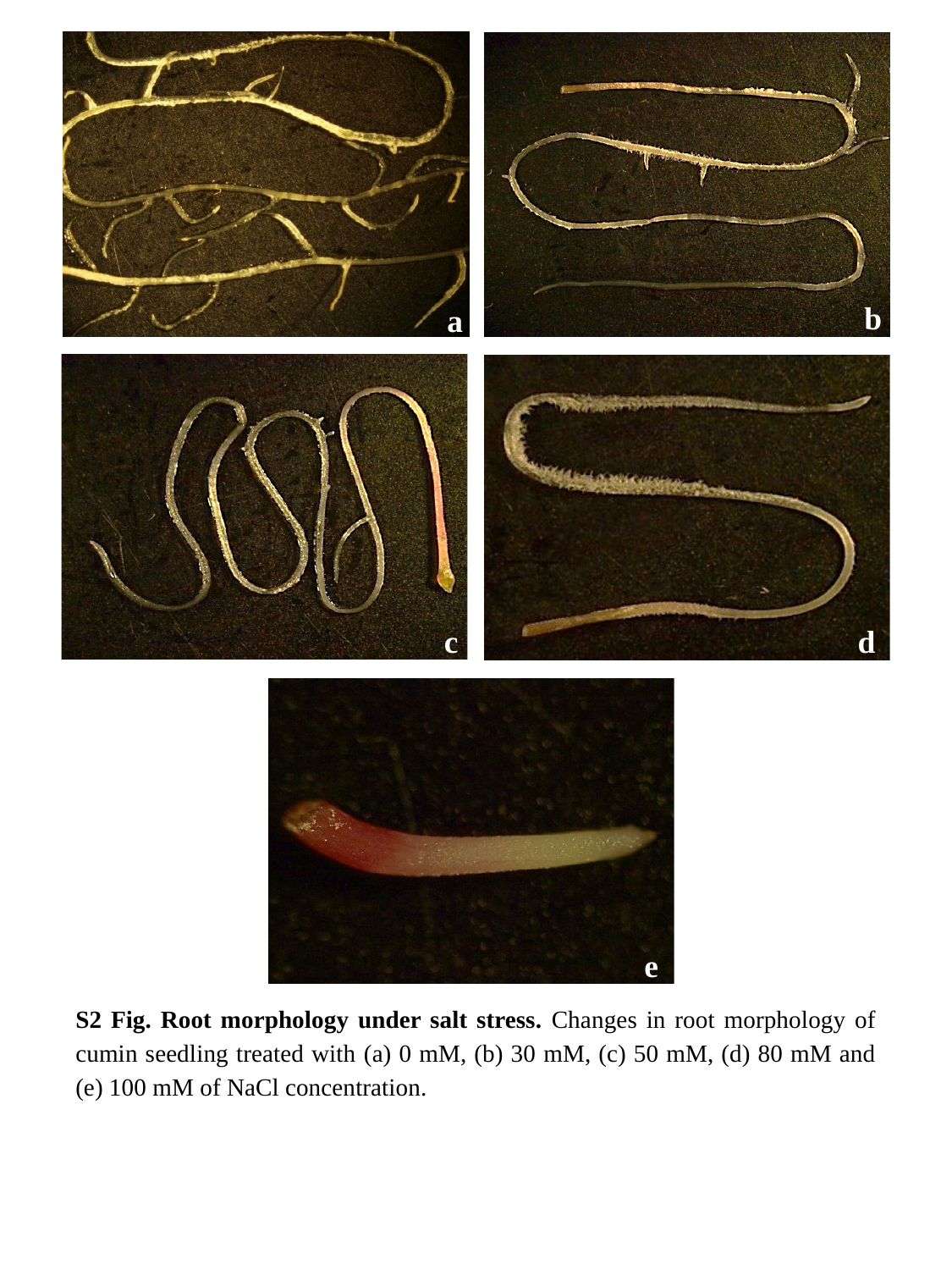

a
b
c
d
e
S2 Fig. Root morphology under salt stress. Changes in root morphology of cumin seedling treated with (a) 0 mM, (b) 30 mM, (c) 50 mM, (d) 80 mM and (e) 100 mM of NaCl concentration.
