# Supplementary material for: Physio-Biochemical Composition and Untargeted Metabolomics of Cumin (Cuminum cyminum L.) Make It Promising Functional Food and Help in Mitigating Salinity Stress
Source: PLoS One. 2015 Dec 7;10(12):e0144469. doi: 10.1371/journal.pone.0144469 (PMC4671573; doi:10.1371/journal.pone.0144469)
Supplement: S3 Fig — Chlorophyll content (S3a), amino acid (S3b), total soluble sugar (S3c), phenolic and flavonoid contents (S3d) of cumin seedling grown under salinity stress. Means ± SE followed by similar letters are significantly different at P<0.05. (PPTX) [file pone.0144469.s003.pptx]

## Slide 1
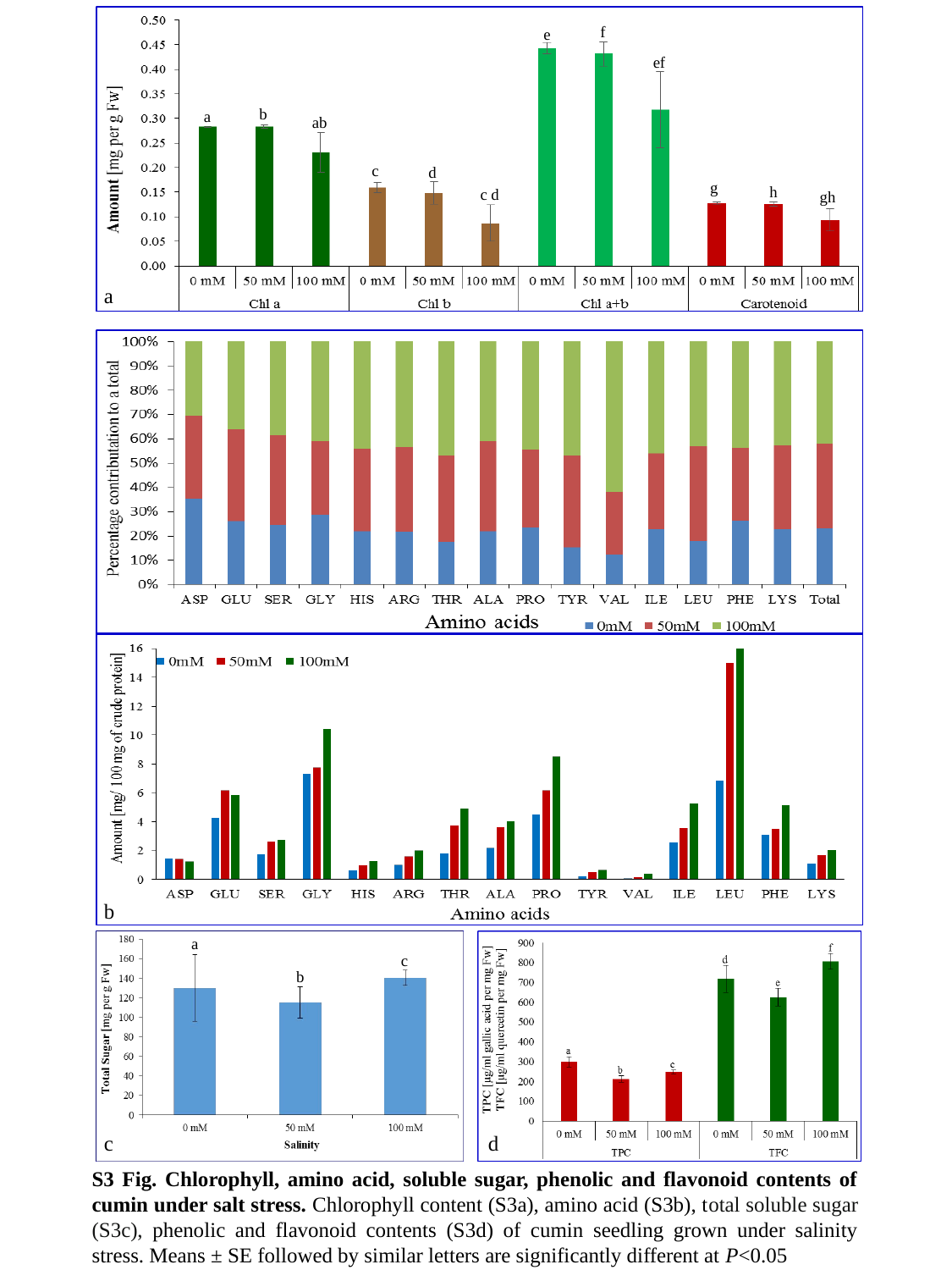

f
e
ef
b
a
ab
c
d
g
h
c d
gh
a
c
b
a
b
c d
S3 Fig. Chlorophyll, amino acid, soluble sugar, phenolic and flavonoid contents of cumin under salt stress. Chlorophyll content (S3a), amino acid (S3b), total soluble sugar (S3c), phenolic and flavonoid contents (S3d) of cumin seedling grown under salinity stress. Means ± SE followed by similar letters are significantly different at P<0.05
