# Supplementary material for: Physio-Biochemical Composition and Untargeted Metabolomics of Cumin (Cuminum cyminum L.) Make It Promising Functional Food and Help in Mitigating Salinity Stress
Source: PLoS One. 2015 Dec 7;10(12):e0144469. doi: 10.1371/journal.pone.0144469 (PMC4671573; doi:10.1371/journal.pone.0144469)
Supplement: S4 Fig — Total antioxidant (S4a) and DPPH inhibition (S4b) activity of cumin seedling under varying salinity stress. Value represents the mean ± SE. (PPT) [file pone.0144469.s004.ppt]

## Slide 1
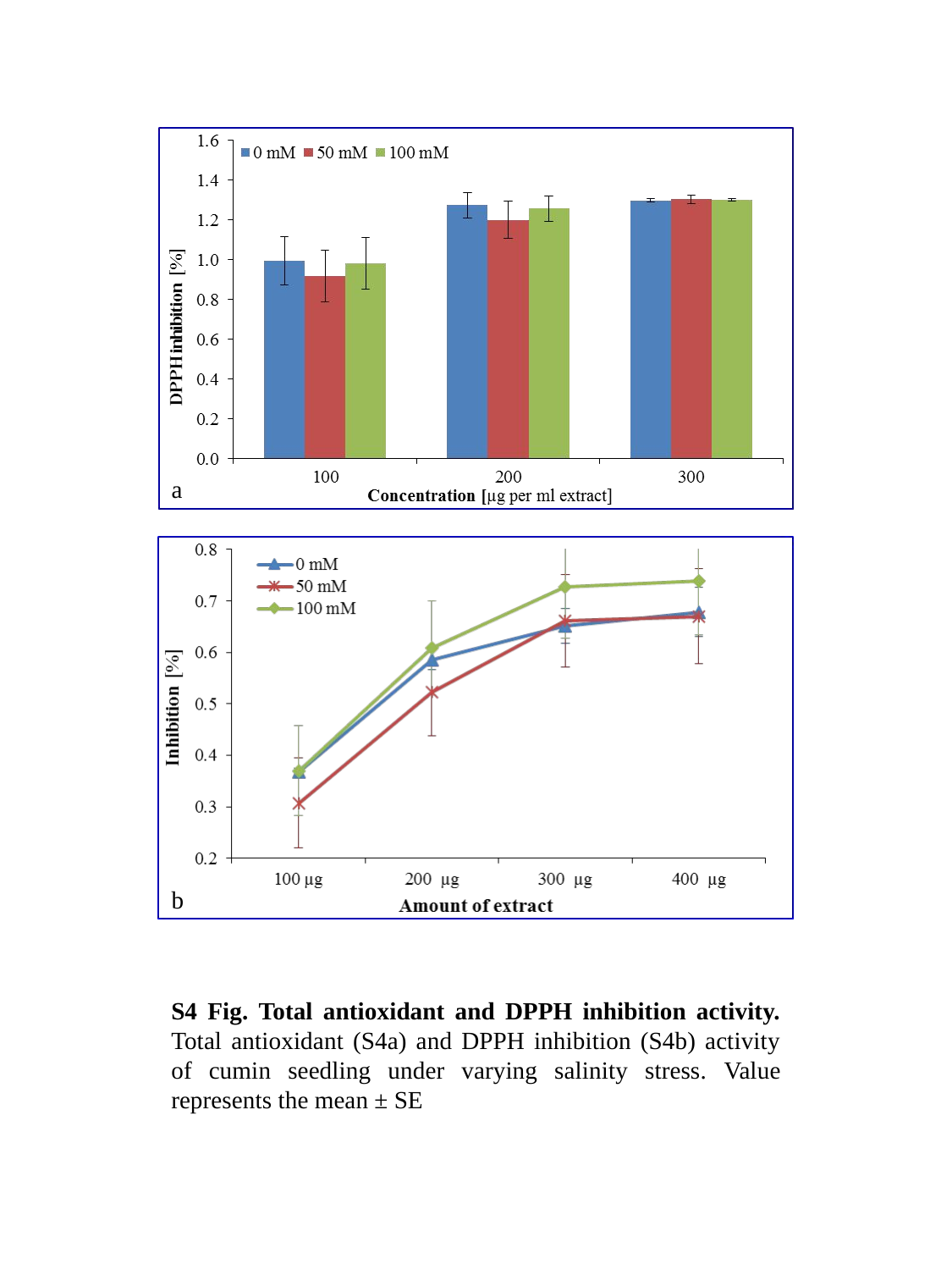

a
b
S4 Fig. Total antioxidant and DPPH inhibition activity. Total antioxidant (S4a) and DPPH inhibition (S4b) activity of cumin seedling under varying salinity stress. Value represents the mean ± SE
