# Supplementary material for: Physio-Biochemical Composition and Untargeted Metabolomics of Cumin (Cuminum cyminum L.) Make It Promising Functional Food and Help in Mitigating Salinity Stress
Source: PLoS One. 2015 Dec 7;10(12):e0144469. doi: 10.1371/journal.pone.0144469 (PMC4671573; doi:10.1371/journal.pone.0144469)
Supplement: S5 Fig — Value represents the mean ± SE. (PPTX) [file pone.0144469.s005.pptx]

## Slide 1
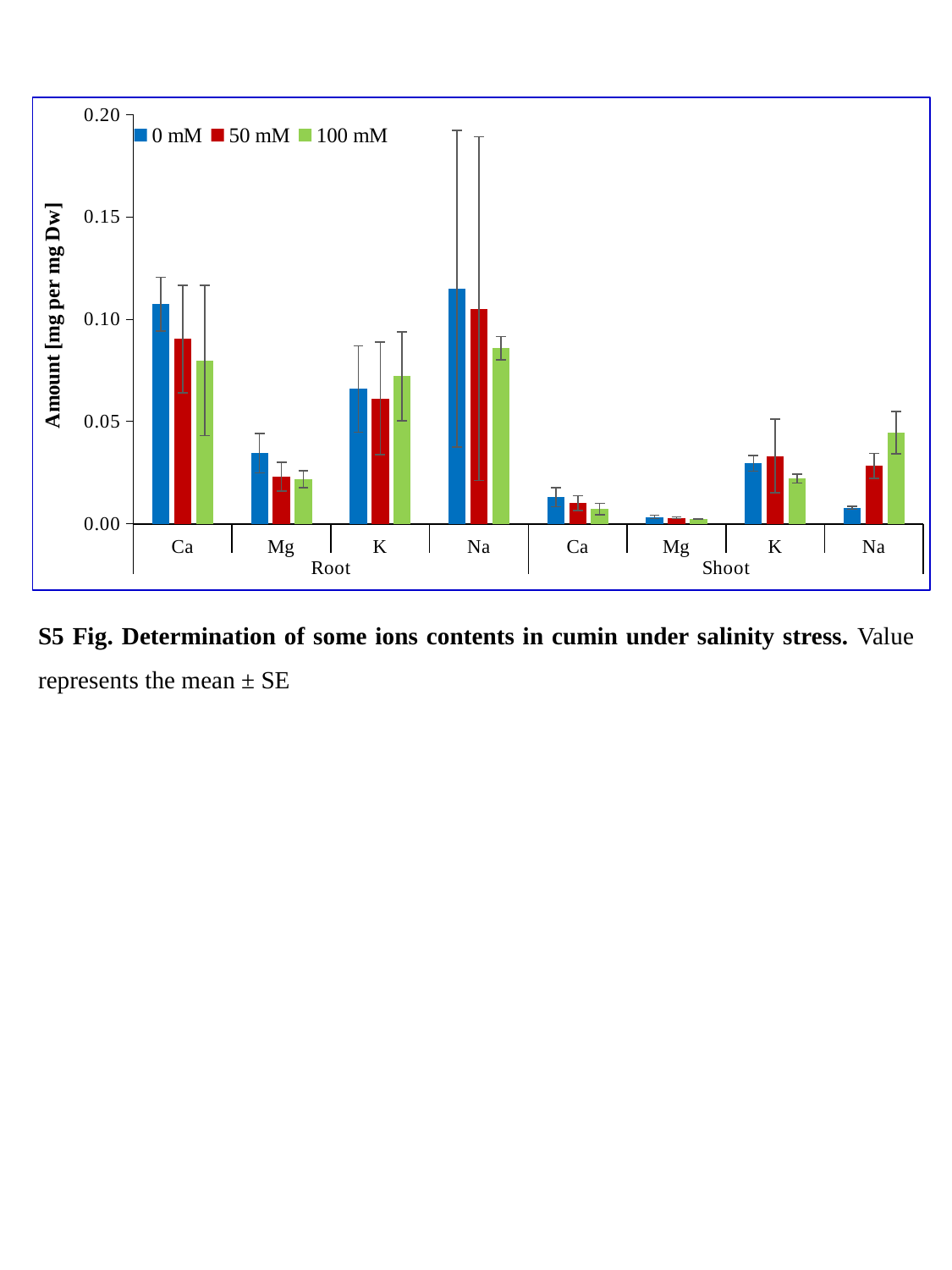

### Chart
| Category | 0 mM | 50 mM | 100 mM |
|---|---|---|---|
| Ca | 0.1072852775 | 0.090381136 | 0.079854957 |
| Mg | 0.03465829962613355 | 0.02313266105260383 | 0.021858462263514407 |
| K | 0.06596372746546353 | 0.061355107957490274 | 0.07214995780906591 |
| Na | 0.11491005825529245 | 0.1051677715308023 | 0.08584938167885689 |
| Ca | 0.013139028284408827 | 0.01015406340353272 | 0.0072436091298701995 |
| Mg | 0.003468717854707893 | 0.0030318740896846646 | 0.0024022301330468556 |
| K | 0.029595702669784833 | 0.0331841094129462 | 0.02216979251553612 |
| Na | 0.007938751907534335 | 0.02839436137638453 | 0.044578546628101706 |S5 Fig. Determination of some ions contents in cumin under salinity stress. Value represents the mean ± SE
